# Psychological health conditions of ART treated infertile couples: a 4-years prospective study

**DOI:** 10.3389/fpsyg.2025.1616754

**Published:** 2025-08-07

**Authors:** Maria Francesca Cattaneo Della Volta, Federica Vallone, Pasquale Dolce, Maria Clelia Zurlo

**Affiliations:** ^1^Dynamic Psychology Laboratory, Department of Political Sciences, University of Naples Federico II, Naples, Italy; ^2^Department of Humanities, University of Naples Federico II, Naples, Italy; ^3^Biostatistics and Clinical Trial Methodology Unit, Clinical Research Center DEMeTra, Department of Translational Medical Science, University of Naples Federico II, Naples, Italy

**Keywords:** infertility, prospective study, ART treatments, adoption, psychological health

## Abstract

**Introduction:**

Infertility literature has well-demonstrated the psychological burden of long-term Assisted Reproductive Technologies treatments and repeated failures, but there is a lack of studies following couples over time, starting from the beginning of the infertility/treatment path, so allowing a greater understanding of the potential outcomes couples may go through (Parenthood after Successful Treatments, PST; Parenthood by Adoption, PA; Childless still Pursuing Treatments, CPT; Childless Quitting Treatments, CQT) and, accordingly, exploring the different impact on their psychological health. This prospective study aims at assessing and comparing psychological health reported by male and female partners of infertile couples at two-time points (T1-beginning of medical treatments; T2-after 4 years), grouping them by the outcome.

**Methods:**

Participants were 108 couples out of 115 couples undergoing infertility treatments recruited in 2018 (T1) available for the follow-up and grouped by the outcome in 2022 (T2). Psychopathological symptoms, measured by SCL-90-R, were assessed at T1 and T2 and compared by outcome groups and sex. Linear Mixed Effect Models were used. Frequencies/percentages of clinically relevant levels of psychopathological symptoms were also explored.

**Results:**

Statistically significant Group-by-Time interaction effects were found. At T1, members of infertile couples reported a substantially comparable psychological portrait, while significant changes at T2 according to the prospective outcome groups for all SCL-90-R subscales were found. Some specificities in changes by sex also emerged. Overall, findings showed a significant increase in psychopathological symptoms among both members of couples Childless and Pursuing Treatments (CPT) and a decreasing trend among members of couples who achieved Parenthood after Successful Treatments (PST) or by Adoption (PA), mainly among men. Considering clinically relevant psychopathological symptoms, data confirmed the abovementioned trends, yet further sex specificities in clinical profiles were found. At T2, CPT couples showed noteworthy increases in clinical obsessive-compulsive symptoms (among women), hostility and interpersonal sensitivity (among men), and anxiety, depression, and paranoid ideation (across sex). Differently, somatization increased over time in women of all prospective outcome groups.

**Discussion:**

Findings can be used to develop tailored evidence-based interventions to promote psychological health and prevent disease escalation during/after infertility treatments.

## 1 Introduction

Infertility is a medical condition that implies no clinical pregnancy after 12 months of regular sexual relations without contraception, resulting from physical and/or psychological impairments in a person's ability to reproduce as an individual or with a male/female partner (Zegers-Hochschild et al., 2017). It affects about 10% to 15% of reproductive-aged couples worldwide (Kamel, 2010; World Health Organization [WHO], 2023), and the prevalence rate in women increases by 0.37% per year (Sun et al., 2019).

Infertility literature has well-demonstrated the psychological burden of infertility experience (Öztürk et al., 2021; Zurlo et al., 2017, 2020a,b), mainly among women who are indeed forefront facing medical interventions, undergo the bulk of invasive procedures (the nature, intensity, and intrusiveness of the medical treatments), and experience disruption in their schedules to accommodate rigid treatment regimens (Gameiro et al., 2013; Schaller et al., 2016; Wischmann, 2020; Yokota et al., 2022). Specifically, research highlighted significantly higher levels of psychopathological symptoms, mainly in terms of anxiety, depression, interpersonal sensitivity among infertile women facing Assisted Reproductive Technologies (ART) treatments if compared with fertile control groups (Lakatos et al., 2017; Noorbala et al., 2009; Vikström et al., 2015; Yusuf, 2016). Accordingly, other studies showed that women suffering from involuntary childlessness reported significantly higher scores on all the subscales of the Symptom Checklist-90-Revised (SCL-90-R) when compared to women in the control group (Dyer et al., 2005).

Since most studies have focused on the experience of women in the understanding that they carry the main burden of infertility treatment path, there is limited information on the experience of men suffering from infertility. Indeed, only a few studies have included male participants, highlighting how infertile men reported significantly higher psychological symptoms than men belonging to the fertile group, especially in terms of depression, anxiety (Cattaneo Della Volta et al., 2022; Johansson et al., 2010; Ozkan et al., 2015; Pasch et al., 2016), interpersonal sensitivity, and hostility (Abdullah et al., 2017).

More recently, further studies have emphasized the negative effects of the length of infertility and repeated treatment failures in exacerbating infertile patients' adverse psychological health outcomes (González-Rodríguez et al., 2020; Kooli et al., 2023; Maroufizadeh et al., 2015; Shakeri et al., 2006; Sohbati et al., 2021; Zurlo et al., 2018, 2023), with psychological suffering worsening within the first 3 years from diagnosis and medical treatment failures (Smith et al., 2009; Zuraida, 2010). In the same direction, several studies highlighted higher rates of discontinuation of fertility treatments (dropout) (Domar et al., 2010; Gameiro et al., 2012) prevalently due to psychological factors (e.g., burden; depression; hopelessness), financial reasons (i.e., depletion of financial resources), demographic factors (e.g., age; educational level), and interpersonal reasons (e.g., lack of social support; divorce) (Ghorbani et al., 2022; Khalili et al., 2012).

However, despite the well-known psychological impairment due to prolonged ART treatments, there is still a need of comparative studies—and even more of prospective studies—examining the psychological impact of treatment success/failure in both male and female partners and following infertile patients over time. Nonetheless, assessing psychological health conditions and potential psychopathological symptoms reported by infertile patients—compared by the outcome (patients achieving Parenthood after Successful Treatments, PST; patients achieving Parenthood by Adoption, PA; patients Childless still Pursuing Treatments, CPT; and patients Childless who have Quitted medical Treatments, CQT) at the baseline and after a period of time—would allow a greater understanding of the phenomenon and provide, accordingly, more tailored multidisciplinary support interventions.

To the best of our knowledge, only a few cross-sectional studies examined infertile patients' psychological health conditions after ART treatments by comparing those who were unsuccessful in achieving parenthood with those who were successful or with those who opted for adoption. These studies highlighted, among unsuccessful patients, an increased risk of reporting anxiety and depression (Verhaak et al., 2007; Yli-Kuha et al., 2010; Zurlo et al., 2023) as well as shame and self-judgment (Galhardo et al., 2011). However, there is also some mixed evidence on the topic. Indeed, some studies highlighted a higher risk for reporting affective disorders (Baldur-Felskov et al., 2013) and state anxiety (Csemiczky et al., 2000) in women giving birth after ART in comparison with those who remained childless after treatment failure, even if recognizing a higher risk for developing other psychological health outcomes among childless women still facing medical treatments. Moreover, further studies even revealed that women who achieved a live birth after ART treatment were at higher risk of developing psychiatric disorders (Munk-Olsen and Agerbo, 2015), mainly depression and post-partum depression (Sejbaek et al., 2015), if compared with women who had not yet given birth to a child and were still pursuing treatments. Furthermore, research also showed no significant differences between patients with successful ART treatments and those who remained childless, with only adoptive parents reporting a lower risk for mental health (Agerbo et al., 2013).

Even fewer studies have been designed as follow-up, so focusing on infertile patients from a prospective view. However, most of them specifically focused on predictors of ART treatments success and didn't explore psychological health conditions, neither compare members of infertile couples by outcome groups (Boivin and Schmidt, 2005; Khalili et al., 2012; Pinborg et al., 2009; Wischmann et al., 2012). In the same direction, most of the few follow-up studies targeting psychological health conditions focused on female infertile patients alone, without considering psychological health conditions reported by male partners (Baldur-Felskov et al., 2013; Bryson et al., 2000; Sejbaek et al., 2015; Vikström et al., 2015). To the best of our knowledge, the only follow-up study assessing the mental health of infertile men and comparing them by the outcome group showed a tendency among childless men to have a more vulnerable mental status than both the fertile control group and those who achieved parenthood after in-vitro fertilization (IVF) treatments (Sydsjö et al., 2015).

Therefore, overall, there is a need to develop further research adopting a prospective design, considering infertility experience as a shared concern, and comparing results by the outcome groups, so gaining a greater understanding of the potential impact of infertility on psychological health conditions of both male and female partners undertaking ART treatments. This approach could, indeed, support the development of tailored and evidence-based counseling interventions to prevent psychopathological symptoms and promote infertile patients' wellbeing among would-be parents during and after ART treatments. The present study aims to target this goal.

Considering the shared nature of infertility experience, the present prospective study targets both male and female partners of infertile couples and aims at assessing and comparing the psychological health conditions of study participants grouping them prospectively by the outcome (patients achieving Parenthood after Successful Treatments, PST; patients achieving Parenthood by Adoption, PA; patients Childless still Pursuing Treatments, CPT; patients Childless who have Quitted Treatments, CQT) at T1 (at the beginning of infertility treatments, in 2018) and at T2 (after 4 years, in 2022).

Given the scarcity of comparative and prospective studies in this field, the following research question, rather than a formal hypothesis, has been proposed and originally tested:

Research Question. Are there any differences in changes over a period of 4 years (between T1 and T2) in levels of psychopathological symptoms reported by male and female partners of infertile couples according to the prospective outcome groups, i.e.: patients achieving Parenthood after Successful Treatments (PST), patients achieving Parenthood by Adoption (PA), patients Childless still Pursuing Treatments (CPT), and patients Childless who have Quitted Treatments (CQT)?

## 2 Materials and methods

### 2.1 Participants and sampling

This prospective study targeted 115 infertile couples that were recruited and surveyed at three Centers of Reproductive Medicine of Udine and Naples (Italy) in September 2018 (T1) and that were asked to participate in the follow-up, after 4 years, in September 2022 (T2).

In both times, chairpersons were asked to give the authorization for administering a questionnaire in their centers/agency and, after obtaining their adhesion to the project, infertile couples were directly asked to participate in the study. Afterwards, couples were asked to individually complete a questionnaire lasting 15–20 min (one session), and one of the authors was present to answer any queries raised by participants. All the patients were fully informed about the purpose of the study. They were assured about the confidentiality of the data, and they were informed that the data would be used only for the aim of the research. The project was approved by the Ethical Committee of Psychological Research of the University of Naples Federico II (IRB:34/2019). Research was performed in accordance with the 1964 Helsinki declaration and its later amendments or comparable ethical standards. Every precaution was taken to protect the privacy of participants and the confidentiality of their personal information. Verbal and written informed consent were obtained from each subject prior to participating in the study.

At T1, to be eligible for the study, participants had to meet the following criteria: (a) couples that had been diagnosed with infertility (Male Factor; Female Factor; Combined Factor; Unexplained); (b) infertile couples who were undergoing their first ART treatment; and (c) the agreement by both members of the couple to participate in the study in order to consider couple sharing infertility problems as research unit. Descriptives of socio-demographic characteristics and infertility-related parameters of women and men at T1 are reported in Table 1. All the participants involved in the study reported a diagnosis of primary infertility and were undergoing homologous ART treatments.

**Table 1:** Socio-demographic characteristics and infertility-related parameters at T1 (*N* = 108 couples).

**Socio-demographic characteristics**	**Women**	**Men**	**Couples**
Age (*M ± SD*)	34.18 ± 3.52	36.70 ± 2.95	
**Educational level [*****n*** **(%)]**			
Primary school	13 (12%)	23 (21.2%)	
High school	61 (56.5%)	53 (49.1%)	
College	34 (31.5%)	32 (29.7%)	
**Employment status [*****n*** **(%)]**			
Unemployed	23 (21.3%)	8 (7.4%)	
Employed	85 (78.7%)	100 (92.6%)	
**Infertility-related parameters**			
Duration of infertility in years (*M ± SD*)			1.09 ± 0.23
**Type of diagnosis [*****n*** **(%)]**			
Male factor			34 (31.5%)
Female factor			42 (38.9%)
Combined factor			16 (14.8%)
Unexplained			16 (14.8%)

At T2, the only inclusion criterion was the agreement by both members of the couple to complete the survey. Of the 115 couples recruited at T1, 108 couples were available to participate in the follow-up at T2.

### 2.2 Measures

The questionnaire was administered at T1 and T2. It included a section dealing with background information and a measurement tool assessing psychological heath conditions.

Background information sheet contained questions on Socio-demographic characteristics, i.e., Age (in years), Educational Level (Primary school; High school; College), and Employment status (Unemployed/Employed), and on infertility-related parameters, i.e., Duration of infertility (in years), number of Previous Treatments and Type of Diagnosis (Male Factor; Female Factor; Combined Factor; Unexplained).

Psychological health conditions were measured by using the SCL-90-R (Derogatis, 1983) Italian version (Prunas et al., 2010) and referring to the cut-off scores to define clinical levels of psychopathological symptoms. The scale comprises 90 items on a 5-point Likert scale ranging from zero (Not at all) to four (Extremely) and divided into nine subscales: Anxiety (10 items, Cronbach's α = 0.84; Cut-off male = 0.91, Cut-off female = 1.31), Depression (13 items, Cronbach's α = 0.87; Cut-off male = 1.08, Cut-off female = 1.62), Somatization (12 items, Cronbach's α = 0.83; Cut-off male = 1.09, Cut-off female = 1.67), Interpersonal Sensitivity (9 items, Cronbach's α = 0.83; Cut-off male = 1.01, Cut-off female = 1.34), Hostility (6 items, Cronbach's α = 0.80; Cut-off male = 1.18, Cut-off female = 1.34), Obsessive-Compulsive (10 items, Cronbach's α = 0.82; Cut-off male = 1.41, Cut-off female = 1.61), Phobic-Anxiety (7 items, Cronbach's α = 0.68; Cut-off male = 0.44, Cut-off female = 0.72), Psychoticism (10 items, Cronbach's α = 0.77; Cut-off male = 0.71, Cut-off female = 0.81), and Paranoid Ideation (6 items, Cronbach's α = 0.76; Cut-off male = 1.00, Cut-off female = 1.67). Standards for scoring and data interpretation are provided in the appendix of the manual (SCL-90-R; Italian version: Prunas et al., 2010). Raw scores were calculated by dividing the sum of the scores of each dimension by the number of items of that dimension. The raw scores were then matched with standardized T-scores by referring to the appropriate reference group by sex (women and men) and age (adolescents, young adults, and adults). A threshold T-score of 65 indicates severe intensity of discomfort and is used—in research and practice—to identify the presence of clinically relevant symptomatology. Therefore, in the present study, the T-score of 65 was used to identify subjects with intense and clinically relevant levels of psychopathology—specifically concerning each of the nine subscales of the SCL-90-R. The two appendices showing the reference group of adult women and adult men, respectively, were used to identify the raw scores matching with the T-scores equal to 65. Cut-off scores and reliabilities were provided by the Italian validation study (Prunas et al., 2010).

### 2.3 Data analyses

Preliminarily, participants were prospectively categorized, at T2, by the following four outcome groups: patients achieving Parenthood after Successful Treatments (PST); patients achieving Parenthood by Adoption (PA); patients Childless still Pursuing Treatments (CPT), and patients Childless who have Quitted Treatments (CQT). Descriptive statistics were computed by the abovementioned outcome groups (PST; PA; CPT; CQT) to summarize the data, including mean ± standard deviation for quantitative variables and frequency (percentage) for categorical variables. The study participants were also compared at baseline (T1) with respect to socio-demographic characteristics, infertility-related parameters, and psychological health conditions, thus exploring the presence of potential baseline imbalances. Specifically, ANOVAs was used for quantitative variables whereas χ^2^ test was used for categorical variables.

Afterwards, considering the three-level structure of the data (repeated measures—time points—nested within individuals—women and men—who were, in turn, nested within couples), to respond to our Research Question linear mixed-effects modeling was employed. The model included a random intercept for each couple and each subject within the couple to capture the within-subject and within-couple correlations. First, the fixed effects included also the interaction terms between group, time, and sex to account for the potential influence of these factors on the outcome variable. If an interaction was not statistically significant, the corresponding term was removed from the model. Model parameters were estimated by optimizing the restricted maximum likelihood (REML) criterion. The results of the mixed-effects model, including the estimated coefficients and *p*-values, were summarized in tables to provide a comprehensive overview of the fixed effects. In addition, plots were generated to display the measurements over time for each outcome group, separated by women and men. This visualization helped to illustrate the trends and differences across groups and time points.

Finally, the study variables, i.e., psychopathological symptoms measured by the Italian version of the SCL-90-R (Prunas et al., 2010), were dichotomized into low (< T-score cut-off) and high levels (≥ T-score cut-off; clinically relevant) referring to the clinical T-score cut-off points of the nine subscales (Somatization, Anxiety, Depression, Hostility, Interpersonal Sensitivity, Obsessive-Compulsive, Paranoid Ideation, Phobic-Anxiety, and Psychoticism). Frequencies and percentages of infertile patients reporting low and high (clinically relevant) levels of psychopathological symptoms were calculated. All the statistical analyses were carried out using R statistical software. Mixed-effects modeling was employed using the lmer function from the lme4 package in R. All statistical tests were performed at a significance level α = 0.05.

## 3 Results

### 3.1 Preliminary findings

In 2022 (T2), due to the prospective nature of the study, if one or both partners didn't complete the survey, the couple was not included in the final dataset. At T2, 6 couples out of 115 have chosen to not participate in the survey and one couple was excluded because the male member deceased. Thus, 108 out of 115 (93.9%) couples were available for the follow-up after 4 years.

Specifically, 108 couples (108 male, 108 female) participated in the study (T2), of whom 42.6% achieved Parenthood after Successful Treatments (PST), 18.5% quitted medical treatments and achieved Parenthood by Adoption (PA), 38.9% were Childless and still Pursuing medical Treatments (CPT), and no Childless couples who Quitted Treatments (CQT) were found (Table 2).

**Table 2:** Prospective outcome groups and number of medical treatments at T2 (*N* = 108 couples).

	**CPT couples**	**PST couples**	**PA couples**
Treatment outcome [*n* (%)]	42 (38.9%)	46 (42.6%)	20 (18.5%)
Repeated medical treatments (*M ± SD*) [range]	7.24 ± 0.93 [6–9]	3.70 ± 0.81 [2–5]	2.95 ± 0.99 [2–4]

CPT, childless still pursuing treatments; PST, parenthood after successful treatments; PA, parenthood by adoption.

Additionally, data indicates that the participants were comparable at T1, suggesting there is no imbalance between the prospective outcome groups in baseline variables that may influence study results. Specifically, considering socio-demographic characteristics and infertility-related parameters, no statistically significant differences are observed between the prospective study groups, and data are displayed in Table 3.

**Table 3:** Socio-demographic characteristics and infertility-related parameters of study participants at baseline (T1) by outcome groups.

**Socio-demographic characteristics**	**Women**					**Men**				
	**Total sample women (*****n*** = **108)**	**CPT (*****n*** = **42)**	**PST (*****n*** = **46)**	**PA (*****n*** = **20)**	***p***	**Total sample men (*****n*** = **108)**	**CPT (*****n*** = **42)**	**PST (*****n*** = **46)**	**PA (*****n*** = **20)**	***p***
Age (*M ± SD*)	34.18 ± 3.52	33.64 ± 3.57	34.33 ± 3.64	34.95 ± 3.09	0.368^a^	36.70 ± 2.95	36.12 ± 3.13	36.72 ± 2.96	37.90 ± 2.22	0.084^a^
**Educational level [*****n*** **(%)]**										
Primary school	13 (12.0%)	6 (46.2%)	4 (30.7%)	3 (23.1%)		23 (21.2%)	11 (47.9%)	7 (30.4%)	5 (21.7%)	
High school	61 (56.5%)	26 (42.6%)	28 (45.9%)	7 (11.5%)	0.220^b^	53 (49.1%)	21 (39.6%)	25 (47.2%)	7 (13.2%)	0.449^b^
College	34 (31.5%)	10 (29.4%)	14 (41.2%)	10 (29.4%)		32 (29.7%)	10 (31.3%)	14 (43.7%)	8 (25.0%)	
**Employment status [*****n*** **(%)]**										
Unemployed	23 (21.3%)	7 (30.4%)	12 (52.2%)	4 (17.4%)	0.552^b^	8 (7.4%)	4 (50.0%)	4 (50.0%)	0 (0.0%)	0.371^b^
Employed	85 (78.7%)	35 (41.2%)	34 (40.0%)	16 (18.8%)		100 (92.6%)	38 (38.0%)	42 (42.0%)	20 (20.0%)	
**Couples**					
**Infertility-related parameters**	**Total couples (*****n*** = **108)**	**CPT (*****n*** = **42)**	**PST (*****n*** = **46)**	**PA (*****n*** = **20)**	***P***
Duration of infertility in years (*M* ±*SD*)	1.09 ± 0.23	1.13 ± 0.28	1.06 ± 0.16	1.10 ± 0.21	0.420^a^
**Type of diagnosis [*****n*** **(%)]**					
Male factor	34 (31.5%)	14 (41.2%)	14 (41.2%)	6 (17.6%)	
Female factor	42 (38.9%)	14 (33.3%)	20 (47.6%)	8 (19.1%)	0.446^b^
Combined factor	16 (14.8%)	4 (25.0%)	8 (50.0%)	4 (25.0%)	
Unexplained	16 (14.8%)	10 (62.5%)	4 (25.0%)	2 (12.5%)	

CPT, childless still pursuing treatments; PST, parenthood after successful treatments; PA, parenthood by adoption. Values are presented as Mean ± SD. *p*-values were computed performing mixed effects modeling. Differences are calculated by ANOVAs (mean ± standard deviations)^a^ and Cross-tabulations and χ^2^-Analyses [*N* (%)]^b^.

This baseline balance is also confirmed when considering participants' psychological health conditions (*F* values *range* from 0.033 for *Somatization* to 2.396 for *Hostility*; all *p* values > 0.05).

### 3.2 Research question: findings

Mean scores (and standard deviations) of the SCL-90-R subscales at T1 and at T2 and findings from Linear Mixed-Effect Model analyses are reported, according to sex and outcome groups, in three convenience tables differentiated by psychopathological symptoms (Tables 4–6).

**Table 4:** Anxiety, depression and somatization: means, standard deviations and findings from linear mixed-effects models by sex and study stages.

	**Women**			**Men**			**Main effects (group, time, sex, interactions)**					
	*T*_1_	*T*_2_		*T*_1_	*T*_2_		***Group***	***Time***	***Sex Men vs. Women***	Group^*^Time Interact.	Sex^*^Group Interact.	Sex^*^Time Interact.
	**M** ±**SD**	**M** ±**SD**		**M** ±**SD**	**M** ±**SD**		β	β	β			
			***p-value***			***p-value***	**[** ***p-value*** **]**	**[** ***p-value*** **]**	**[** ***p-value*** **]**	***p-value***	***p-value***	***p-value***
**Anxiety**												
CPT (*n* = 42)	1.27 ± 0.21	1.35 ± 0.20	*** < 0.001***	0.88 ± 0.04	0.93 ± 0.04	*** < 0.001***	/	/	−0.37 **[***** < 0.001*****]**	*** < 0.001***	0.263	0.939
PST (*n* = 46)	1.23 ± 0.19	1.17 ± 0.25	*** < 0.001***	0.87 ± 0.05	0.83 ± 0.04	*** < 0.001***						
PA (*n* = 20)	1.24 ± 0.20	1.20 ± 0.18	***0.009***	0.91 ± 0.04	0.87 ± 0.03	***0.009***						
*CPT vs. PST* [*p-value*]	[0.241]	**[** *** < 0.001*** **]**		[0.241]	**[** *** < 0.001*** **]**							
*CPT vs. PA* [*p-value*]	[0.946]	**[** *** < 0.001*** **]**		[0.946]	**[** *** < 0.001*** **]**							
**Depression**												
CPT (*n* = 42)	1.54 ± 0.15	1.62 ± 0.17	*** < 0.001***	1.20 ± 0.30	1.43 ± 0.30	*** < 0.001***	/	/	/	*** < 0.001***	0.229	***0.020***
PST (*n* = 46)	1.53 ± 0.16	1.53 ± 0.16	0.333	1.30 ± 0.34	1.05 ± 0.14	*** < 0.001***						
PA (*n* = 20)	1.52 ± 0.16	1.49 ± 0.15	0.127	1.24 ± 0.33	1.05 ± 0.16	*** < 0.001***						
*CPT vs. PST* [*p-value*]	[0.218]	**[** *** < 0.001*** **]**		[0.218]	**[** *** < 0.001*** **]**							
*CPT vs. PA* [*p-value*]	[0.900]	**[** *** < 0.001*** **]**		[0.900]	**[** *** < 0.001*** **]**							
**Somatization**												
CPT (*n* = 42)	1.59 ± 0.17	1.64 ± 0.18	*** < 0.001***	1.06 ± 0.11	1.10 ± 0.14	0.115	/	/	/	***0.048***	0.088	***0.002***
PST (*n* = 46)	1.61 ± 0.15	1.65 ± 0.15	***0.045***	1.06 ± 0.11	1.03 ± 0.08	0.170						
PA (*n* = 20)	1.62 ± 0.14	1.69 ± 0.13	***0.016***	1.04 ± 0.08	1.02 ± 0.08	0.982						
*CPT vs. PST* [*p-value*]	[0.565]	[0.115]		[0.565]	[0.115]							
*CPT vs. PA* [*p-value*]	[0.841]	[0.462]		[0.841]	[0.462]							

CPT, childless still pursuing treatments; PST, parenthood after successful treatments; PA, parenthood by adoption. Values are presented as Mean ± SD. *p*-values were computed performing mixed effects modeling. ^*^ refer to interactions. Statistically significant values are highlighted in bold and italics.

**Table 5:** Hostility, interpersonal-sensitivity, obsessive compulsive symptoms: means, standard deviations and findings from linear mixed-effects models by sex and study stages.

	**Women**			**Men**			**Main effects (group, time, sex, interactions)**					
	**T**_1_	**T**_2_		**T**_1_	**T**_2_		***Group***	***Time***	***Sex Men vs. Women***	Group^*^Time Interact.	Sex^*^Group Interact.	Sex^*^Time Interact.
	**M** ±**SD**	**M** ±**SD**		**M** ±**SD**	**M** ±**SD**		β	***B***	β			
			***p-value***			***p-value***	**[** ***p-value*** **]**	**[** ***p-value*** **]**	**[** ***p-value*** **]**	***p-value***	***p-value***	***p-value***
**Hostility**												
CPT (*n* = 42)	1.27 ± 0.12	1.28 ± 0.11	*** < 0.001***	1.15 ± 0.08	1.26 ± 0.09	*** < 0.001***	/	/	−0.11 **[***** < 0.001*****]**	*** < 0.001***	0.064	0.392
PST (*n* = 46)	1.30 ± 0.10	1.29 ± 0.10	***0.010***	1.19 ± 0.10	1.15 ± 0.06	***0.010***						
PA (*n* = 20)	1.30 ± 0.11	1.29 ± 0.10	***0.001***	1.19 ± 0.09	1.12 ± 0.02	***0.001***						
*CPT vs. PST* [*p-value*]	**[** ***0.029*** **]**	**[** *** < 0.001*** **]**		**[** ***0.029*** **]**	**[** *** < 0.001*** **]**							
*CPT vs. PA* [*p-value*]	**[** ***0.049*** **]**	**[** *** < 0.001*** **]**		**[** ***0.049*** **]**	**[** *** < 0.001*** **]**							
**Interpersonal-Sensitivity**												
CPT (*n* = 42)	1.24 ± 0.11	1.25 ± 0.12	*0.020*	1.00 ± 0.06	1.03 ± 0.07	0.527	/	/	/	*** < 0.001***	0.680	***0.003***
PST (*n* = 46)	1.24 ± 0.12	1.23 ± 0.12	0.060	1.04 ± 0.09	0.96 ± 0.09	*** < 0.001***						
PA (*n* = 20)	1.24 ± 0.13	1.24 ± 0.11	0.526	1.01 ± 0.10	0.95 ± 0.10	0.057						
*CPT vs. PST* [*p-value*]	[0.242]	**[** ***0.007*** **]**		[0.242]	**[** ***0.007*** **]**							
*CPT vs. PA* [*p-value*]	[0.619]	**[** ***0.065*** **]**		[0.619]	**[** ***0.065*** **]**							
**Obssessive-Compulsive**												
CPT (*n* = 42)	1.50 ± 0.10	1.59 ± 0.12	*** < 0.001***	1.37 ± 0.06	1.38 ± 0.06	*** < 0.001***	/	/	/	*** < 0.001***	0.432	*** < 0.001***
PST (*n* = 46)	1.51 ± 0.13	1.49 ± 0.12	0.632	1.37 ± 0.08	1.34 ± 0.07	*** < 0.001***						
PA (*n* = 20)	1.51 ± 0.13	1.50 ± 0.12	0.532	1.39 ± 0.07	1.34 ± 0.03	*** < 0.001***						
*CPT vs. PST* [*p-value*]	[0.583]	**[** *** < 0.001*** **]**		[0.583]	**[ < ** ***0.001*** **]**							
*CPT vs. PA* [*p-value*]	[0.411]	**[** *** < 0.001*** **]**		[0.411]	**[** *** < 0.001*** **]**							

CPT, childless still pursuing treatments; PST, parenthood after successful treatments; PA, parenthood by adoption. Values are presented as Mean ± SD. *p*-values were computed performing mixed effects modeling. ^*^ refer to interactions. Statistically significant values are highlighted in bold and italics.

**Table 6:** Paranoid ideation, phobic anxiety and psychoticism: means, standard deviations and findings from linear mixed-effects models by sex and study stages.

	**Women**			**Men**			**Main effects (group, time, sex, interactions)**					
	*T*_1_	*T*_2_		*T*_1_	*T*_2_		**Group**	**Time**	**Sex Men vs. Women**	Group^*^Time Interact.	Sex^*^Group Interact.	Sex^*^Time Interact.
	**M** ±**SD**	**M** ±**SD**		**M** ±**SD**	**M** ±**SD**		β	***B***	β			
			***p-value***			***p-value***	**[** ***p-value*** **]**	**[** ***p-value*** **]**	**[** ***p-value*** **]**	***p-value***	***p-value***	***p-value***
**Paranoid ideation**												
CPT (*n* = 42)	1.48 ± 0.16	1.54 ± 0.18	*** < 0.001***	0.95 ± 0.09	1.06 ± 0.17	*** < 0.001***	/	/	−0.54 **[***** < 0.001*****]**	*** < 0.001***	0.272	0.689
PST (*n* = 46)	1.48 ± 0.16	1.45 ± 0.18	*** < 0.001***	0.93 ± 0.07	0.88 ± 0.03	*** < 0.001***						
PA (*n* = 20)	1.48 ± 0.15	1.46 ± 0.16	***0.038***	0.93 ± 0.06	0.90 ± 0.03	***0.038***						
*CPT vs. PST* [*p-value*]	[0.496]	**[** *** < 0.001*** **]**		[0.496]	**[** *** < 0.001*** **]**							
*CPT vs. PA* [*p-value*]	[0.712]	**[** *** < 0.001*** **]**		[0.712]	**[** *** < 0.001*** **]**							
**Phobic-Anxiety**												
CPT (*n* = 42)	0.68 ± 0.10	0.70 ± 0.10	*** < 0.001***	0.40 ± 0.03	0.44 ± 0.05	*** < 0.001***	/	/	−0.28 **[***** < 0.001*****]**	*** < 0.001***	0.720	0.290
PST (*n* = 46)	0.69 ± 0.11	0.71 ± 0.10	0.611	0.42 ± 0.06	0.40 ± 0.04	0.611						
PA (*n* = 20)	0.69 ± 0.12	0.70 ± 0.11	0.689	0.42 ± 0.07	0.40 ± 0.05	0.689						
*CPT vs. PST* [*p-value*]	[0.265]	[0.259]		[0.265]	[0.259]							
*CPT vs. PA* [*p-value*]	[0.359]	[0.216]		[0.359]	[0.216]							
**Psychoticism**												
CPT (*n* = 42)	0.61 ± 0.14	0.62 ± 0.14	*** < 0.001***	0.54 ± 0.07	0.57 ± 0.10	*** < 0.001***	/	/	−0.07 **[***** < 0.001*****]**	*** < 0.001***	0.772	0.879
PST (*n* = 46)	0.62 ± 0.14	0.61 ± 0.15	*** < 0.001***	0.57 ± 0.05	0.54 ± 0.05	*** < 0.001***						
PA (*n* = 20)	0.61 ± 0.15	0.60 ± 0.15	***0.004***	0.52 ± 0.03	0.50 ± 0.03	***0.004***						
*CPT vs. PST* [*p-value*]	[0.267]	[0.275]		[0.267]	[0.275]							
*CPT vs. PA* [*p-value*]	[0.692]	**[** ***0.049*** **]**		[0.692]	**[** ***0.049*** **]**							

CPT, childless still pursuing treatments; PST, parenthood after successful treatments; PA, parenthood by adoption. Values are presented as Mean ± SD. *p*-values were computed performing mixed effects modeling. ^*^ refer to interactions. Statistically significant values are highlighted in bold and italics.

**Figure 1:**
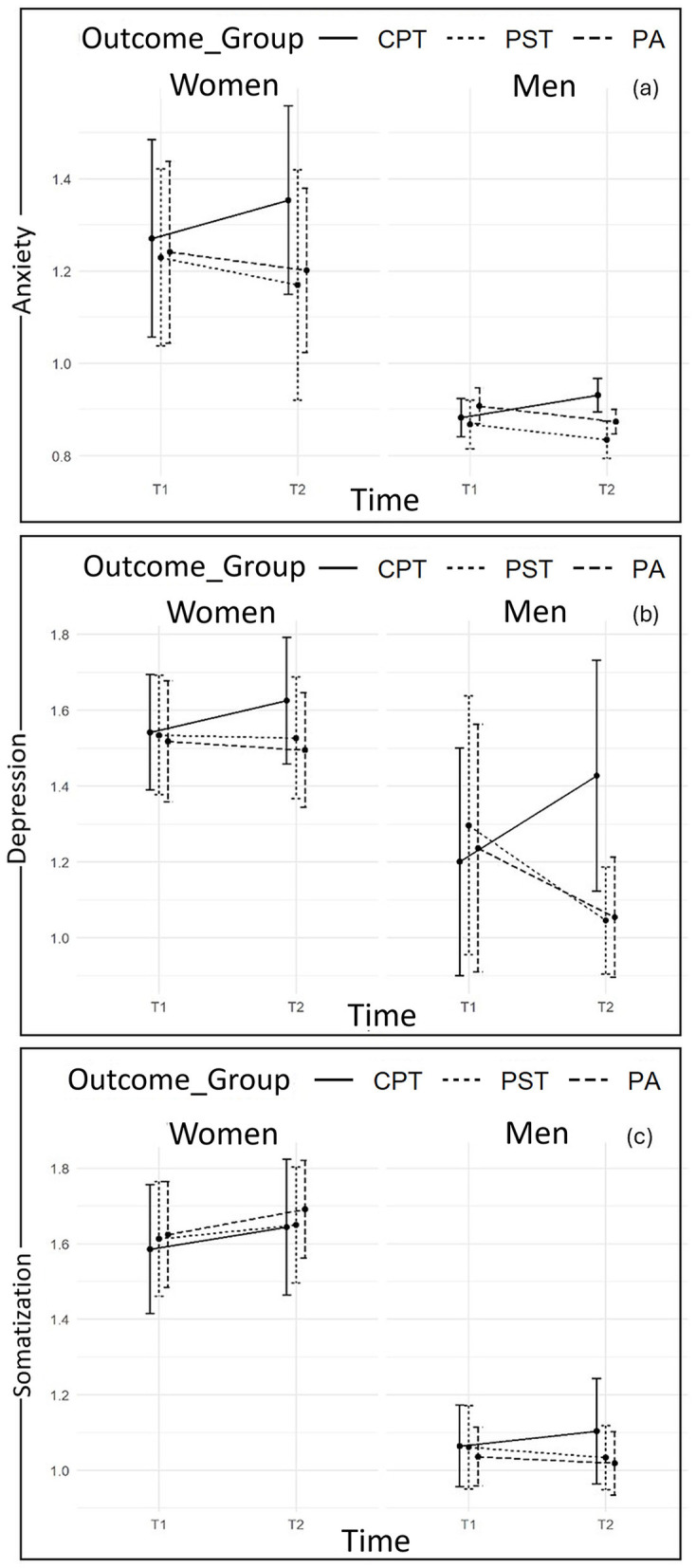
Plots of changes from T_1_ to T_2_ in Anxiety **(a)**, Depression **(b)**, and Somatization **(c)** scores according to sex and outcome groups.

**Figure 2:**
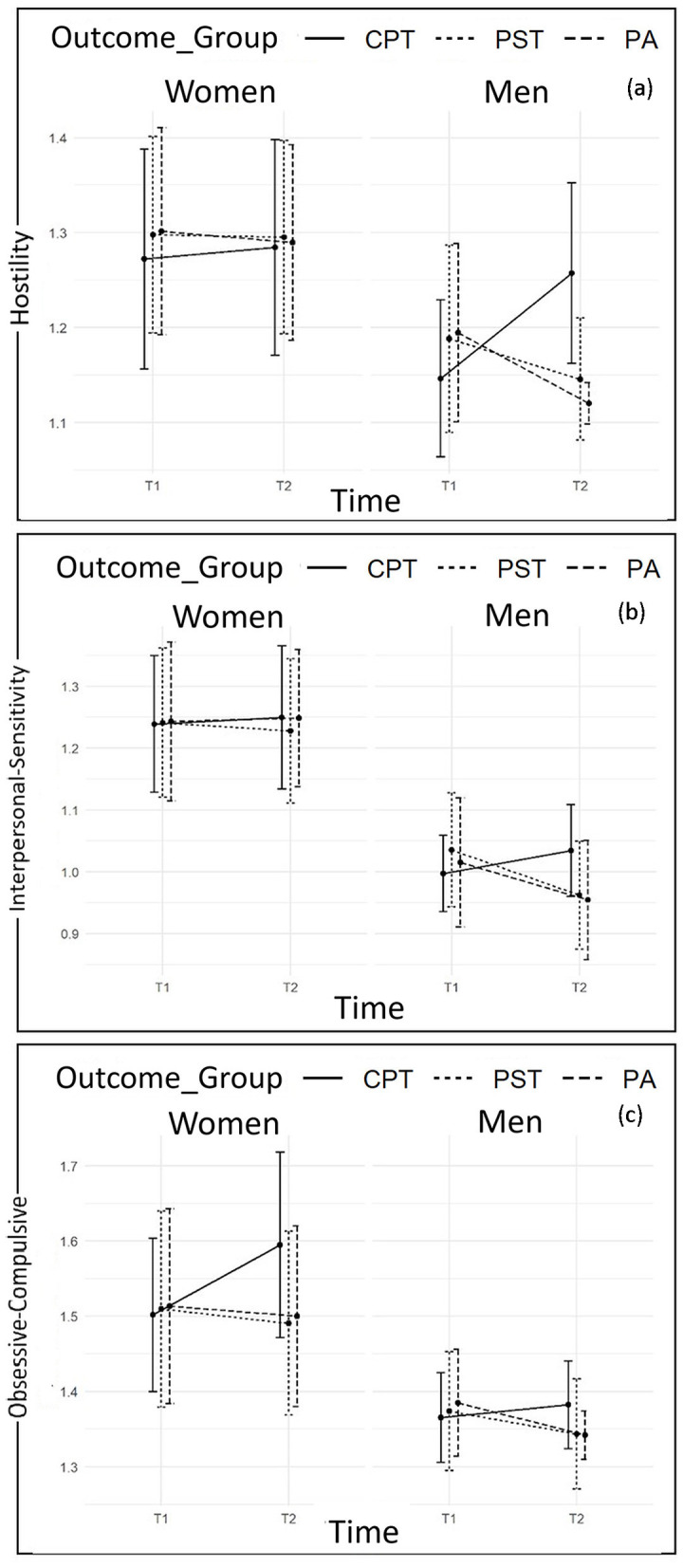
Plots of changes from T_1_ to T_2_ in Hostility **(a)**, Interpersonal-Sensitivity **(b)**, and Obsessive-Compulsive symptoms **(c)** scores according to sex and outcome groups.

**Figure 3:**
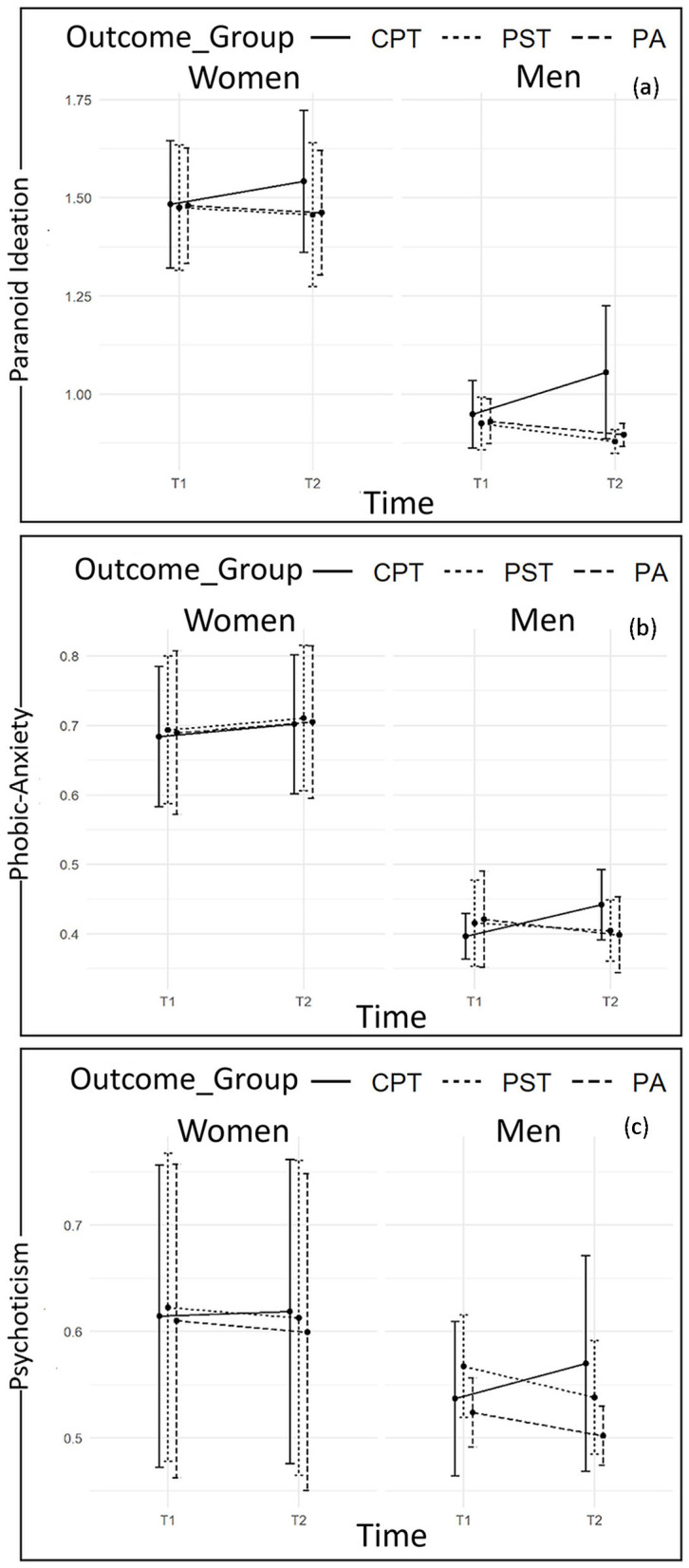
Plots of changes from T1 to T2 in Paranoid Ideation **(a)**, Phobic-Anxiety **(b)**, and Psychoticism **(c)** scores according to sex and outcome groups.

Graphical representations of mean values by sex and outcome groups over time are reported in Figures 1–3.

Data highlights statistically significant Group-by-Time interaction effects for all the models (i.e., for all SCL-90-R subscales), with significant changes between T1 and T2 according to the prospective outcome groups (all *p*-values < 0.05).

At T1, data reveals no statistically significant differences in mean scores/standard deviations of all SCL-90-R subscales by prospective outcome group and by sex, with except for hostility levels which were statistically significantly lower in members of CPT couples if compared with PST and PA groups (*p* < 0.05 for both comparisons; Table 5).

Furthermore, statistically significant Sex-by-Time interaction effects (but no Sex-by-Group interaction effects) are also found, with reference to the models for Depression, Interpersonal-Sensitivity, Somatization, and Obsessive-Compulsive symptoms (all *p*-values < 0.05). Therefore, different trends are observed by sex over time.

Specifically, data at T2 indicates statistically significant increases in levels of Depression and Obsessive-Compulsive symptoms in both members of CPT couples, who have reported statistically significant higher levels of the abovementioned symptoms if compared with members of PST and PA couples. However, women and men of the latter two groups statistically differ at T2, due to the sharper decreasing trend reported by men. Moreover, data at T2 also reveals a statistically significant increase in levels of Interpersonal-Sensitivity only among CPT women, and a statistically significant decrease only among PST men (Figures 1, 2).

Considering Somatization, data reveals a statistically significant increase over time among all the sampled women across the prospective outcome groups, while men followed the same abovementioned trend (increasing scores in CPT and decreasing scores in PST and PA) (Figure 1).

Differently, considering findings from Linear Mixed-Effect Models carried out on the other SCL-90-R subscales, despite women reporting higher scores than men for Anxiety, Hostility, Paranoid Ideation, Phobic Anxiety and Psychoticism, neither Sex-by-Group nor Sex-by-Time interaction effects are found to be statistically significant. Therefore, despite the differences in scores, women and men belonging to the three prospective outcome groups follow the same trend in changes over time. In particular, data at T2 reveals statistically significant increases in levels of Anxiety (Figure 1), Hostility (Figure 2), Paranoid Ideation, and Psychoticism (Figure 3) among both members of CPT couples, who report statistically significant higher levels of the abovementioned symptoms if compared with members of PST and PA couples. The latter groups, conversely, report a statistically significant decreasing trend. However, considering Phobic-Anxiety (Figure 3), the data reveals a similar trend to somatization.

In summary, overall, findings show a significant increase in psychopathological symptoms among members of couples Childless and Pursuing Treatments (CPT) and a decreasing trend among members of couples who achieved Parenthood after Successful Treatments (PST) and/or by Adoption (PA), mainly among men.

### 3.3 Clinically relevant psychological symptoms

Considering frequencies/percentages of clinical levels of psychopathological symptoms according to sex and prospective outcome groups (Tables 7–9), data registered at T1 highlighted several and meaningful information on high prevalence of psychopathological symptoms among women and men beyond the prospective outcome groups. However, when examining data from T1 to T2, findings substantially confirmed the trends emerged from linear mixed-effect models.

**Table 7:** Clinical levels of anxiety, depression and somatization by sex and study stages.

	**Women**		**Men**	
	**T**_1_	**T**_2_	**T**_1_	**T**_2_
	***n*** **(%)**	***n*** **(%)**	***n*** **(%)**	***n*** **(%)**
**Anxiety**				
**CPT (*****n*** = **42)**				
Low (< cut-off)	22 (55.4)	14 (33.3)	33 (78.6)	13 (31.0)
High (clinically relevant)	20 (47.6)	28 (66.7)	9 (21.4)	29 (69.0)
**PST (*****n*** = **46)**				
Low (< cut-off)	27 (58.7)	28 (60.9)	36 (78.3)	44 (95.7)
High (clinically relevant)	19 (41.3)	18 (39.1)	10 (21.7)	2 (4.3)
**PA (*****n*** = **20)**				
Low (< cut-off)	12 (60.0)	13 (65.0)	12 (60.0)	18 (90.0)
High (clinically relevant)	8 (40.0)	7 (35.0)	8 (40.0)	2 (10.0)
**Depression**				
**CPT (*****n*** = **42)**				
Low (< cut-off)	29 (69.0)	14 (33.3)	31 (73.8)	14 (33.3)
High (clinically relevant)	13 (31.0)	28 (66.7)	11 (26.2)	28 (66.7)
**PST (*****n*** = **46)**				
Low (< cut-off)	29 (63.0)	29 (63.0)	30 (65.2)	44 (95.7)
High (clinically relevant)	17 (37.0)	17 (37.0)	16 (34.8)	2 (4.3)
**PA (*****n*** = **20)**				
Low (< cut-off)	13 (65.0)	14 (70.0)	14 (70.0)	19 (95.0)
High (clinically relevant)	7 (35.0)	6 (30.0)	6 (30.0)	1 (5.0)
**Somatization**				
**CPT (*****n*** = **42)**				
Low (< cut-off)	19 (45.2)	13 (31.0)	35 (83.3)	35 (83.3)
High (clinically relevant)	23 (54.8)	29 (69.0)	7 (16.7)	7 (16.7)
**PST (*****n*** = **46)**				
Low (< cut-off)	23 (50.0)	14 (30.4)	41 (89.1)	44 (95.7)
High (clinically relevant)	23 (50.0)	32 (69.6)	5 (10.9)	2 (4.3)
**PA (*****n*** = **20)**				
Low (< cut-off)	10 (50.0)	6 (30.0)	19 (95.0)	19 (95.0)
High (clinically relevant)	10 (50.0)	14 (70.0)	1 (5.0)	1 (5.0)

CPT, childless still pursuing treatments; PST, parenthood after successful treatments; PA, parenthood by adoption.

**Table 8:** Clinical levels of hostility, interpersonal-sensitivity, obsessive compulsive symptoms by sex and study stages.

	**Women**		**Men**	
	*T*_1_	*T*_2_	*T*_1_	*T*_2_
	***n*** **(%)**	***n*** **(%)**	***n*** **(%)**	***n*** **(%)**
**Hostility**				
**CPT (*****n*** = **42)**				
Low (< cut-off)	25 (59.5)	24 (57.1)	35 (83.3)	16 (38.1)
High (clinically relevant)	17 (40.5)	18 (42.9)	7 (16.7)	26 (61.9)
**PST (*****n*** = **46)**				
Low (< cut-off)	25 (54.3)	24 (52.2)	34 (73.9)	43 (93.5)
High (clinically relevant)	21 (45.7)	22 (47.8)	12 (26.1)	3 (6.5)
**PA (*****n*** = **20)**				
Low (< cut-off)	11 (55.0)	11 (55.0)	13 (65.0)	20 (100.0)
High (clinically relevant)	9 (45.0)	9 (45.0)	7 (35.0)	0 (0.0)
**Interpersonal-Sensitivity**				
**CPT (*****n*** = **42)**				
Low (< cut-off)	33 (78.6)	32 (76.2)	36 (85.7)	31 (73.8)
High (clinically relevant)	9 (21.4)	10 (23.8)	6 (14.3)	11 (26.2)
**PST (*****n*** = **46)**				
Low (< cut-off)	36 (78.3)	34 (73.9)	31 (67.4)	40 (87.0)
High (clinically relevant)	10 (21.7)	12 (26.1)	15 (32.6)	6 (13.0)
**PA (*****n*** = **20)**				
Low (< cut-off)	15 (75.0)	15 (75.0)	14 (70.0)	18 (90.0)
High (clinically relevant)	5 (25.0)	5 (25.0)	6 (30.0)	2 (10.0)
**Obsessive-Compulsive**				
**CPT (*****n*** = **42)**				
Low (< cut-off)	33 (78.6)	16 (38.1)	40 (95.2)	40 (95.2)
High (clinically relevant)	9 (24.3)	26 (61.9)	2 (4.8)	2 (4.8)
**PST (*****n*** = **46)**				
Low (< cut-off)	32 (69.6)	31 (67.4)	43 (93.5)	44 (95.7)
High (clinically relevant)	14 (30.4)	15 (32.6)	3 (6.5)	2 (4.3)
**PA (*****n*** = **20)**				
Low (< cut-off)	14 (70.0)	14 (70.0)	19 (95.0)	20 (100.0)
High (clinically relevant)	6 (30.0)	6 (30.0)	1 (5.0)	0 (0.0)

CPT, childless still pursuing treatments; PST, parenthood after successful treatments; PA, parenthood by adoption.

**Table 9:** Clinical levels of paranoid ideation, phobic-anxiety and psychoticism by sex and study stages.

	**Women**		**Men**	
	*T*_1_	*T*_2_	*T*_1_	*T*_2_
	***n*** **(%)**	***n*** **(%)**	***n*** **(%)**	***n*** **(%)**
**Paranoid ideation**				
**CPT (*****n*** = **42)**				
Low (< cut-off)	30 (71.4)	26 (61.9)	34 (81.0)	28 (66.7)
High (clinically relevant)	12 (28.6)	16 (38.1)	8 (19.0)	14 (33.3)
**PST (*****n*** = **46)**				
Low (< cut-off)	35 (76.1)	35 (76.1)	39 (84.8)	46 (100.0)
High (clinically relevant)	11 (23.9)	11 (23.9)	7 (15.2)	0 (0.0)
**PA (*****n*** = **20)**				
Low (< cut-off)	16 (80.0)	16 (80.0)	17 (85.0)	20 (100.0)
High (clinically relevant)	4 (20.0)	4 (20.0)	3 (15.0)	0 (0.0)
**Phobic-Anxiety**				
**CPT (*****n*** = **42)**				
Low (< cut-off)	21 (50.0)	18 (42.9)	39 (92.9)	30 (71.4)
High (clinically relevant)	21 (50.0)	24 (57.1)	3 (7.1)	12 (28.6)
**PST (*****n*** = **46)**				
Low (< cut-off)	23 (50.0)	20 (43.5)	37 (80.4)	36 (78.3)
High (clinically relevant)	23 (50.0)	26 (56.5)	9 (19.6)	10 (21.7)
**PA (*****n*** = **20)**				
Low (< cut-off)	10 (50.0)	9 (45.0)	16 (80.0)	18 (90.0)
High (clinically relevant)	10 (50.0)	11 (55.0)	4 (20.0)	2 (10.0)
**Psychoticism**				
**CPT (*****n*** = **42)**				
Low (< cut-off)	34 (81.0)	34 (81.0)	40 (95.2)	39 (92.9)
High (clinically relevant)	8 (19.0)	8 (19.0)	2 (4.8)	3 (7.1)
**PST (*****n*** = **46)**				
Low (< cut-off)	37 (80.4)	37 (80.4)	45 (97.8)	45 (97.8)
High (clinically relevant)	9 (19.6)	9 (19.6)	1 (2.2)	1 (2.2)
**PA (*****n*** = **20)**				
Low (< cut-off)	16 (80.0)	16 (80.0)	20 (100.0)	20 (100.0)
High (clinically relevant)	4 (20.0)	4 (20.0)	0 (0.0)	0 (0.0)

CPT, childless still pursuing treatments; PST, parenthood after successful treatments; PA, parenthood by adoption.

Considering both women and men belonging to Childless still Pursuing Treatments group (CPT) data highlights an increase from T1 to T2 in frequencies/percentages of clinical levels of Anxiety (women from 47.6 to 66.7%; men from 21.4% to 69%) and Depression (women from 31% to 66.7%; men from 26.2 to 66.7%; Table 7), as well as Paranoid Ideation (women from 28.6 to 38.1%; men from 19% to 33.3%; Table 9). Furthermore, once again in line with findings from Liner Mixed-Effect Models, data indicates an increase from T1 to T2 in the number and percentages of female partners reporting clinical levels of Somatization in all the three prospective outcome groups (CPT: from 54.8 to 69%; PST: from 50 to 69.6%; PA: from 50 to 70%).

However, further sex specificities in clinical profiles are also found. Specifically, only for women belonging to Childless still Pursuing Treatments group (CPT)—and not for the other two prospective outcome groups—data also reveals an increase in frequencies/percentages of clinical levels of Obsessive-Compulsive symptoms (from 24.3% to 61.9%; Table 8).

Considering men, only for those belonging to Childless still Pursuing Treatments group (CPT) data reveals an increase from T1 to T2 in frequencies/percentages of clinical levels of Hostility (from 16.7 to 61.9%) and Interpersonal Sensitivity (from 14.3 to 26.2%; Table 8).

Conversely, for men belonging to both Parenthood after Successful Treatments (PST) and to Parenthood by Adoption (PA) groups, data reveals a substantial decrease in clinical levels of Anxiety (PST from 21.7 to 4.3%; PA from 40 to 10%), Depression (PST from 34.8 to 4.3%; PA from 30 to 5%; Table 7), Hostility (PST from 26.1 to 6.5%; PA from 35 to 0%), Interpersonal Sensitivity (PST from 32.6 to 13%; PA from 30 to 10%; Table 8), and Paranoid Ideation (PST from 15.2 to 0%; PA from 15% to 0%; Table 9).

## 4 Discussion and conclusions

The present prospective study targeted both members of infertile couples and assessed their psychological health conditions when they have undergone their first ART treatment in 2018 (T1), and after 4 years in 2022 (T2), comparing them by the outcomes of infertility treatments, i.e., patients achieving Parenthood after Successful Treatments (PST), patients achieving Parenthood by Adoption (PA), patients Childless still Pursuing Treatments (CPT), and patients Childless Quitting Treatments (CQT).

Firstly, our findings highlighted that - when contacted at T2 (2022)–46 couples (42.6%) out of 108 achieved parenthood after successful treatments (PST), with a range of 2 to 5 treatment cycles. These data substantially align with research underlining that the childbearing rate is 29.7% (Zarinara et al., 2021) and 24.5% (Malizia et al., 2009) within the first treatment cycle, while it becomes 45.2 and 44.9% (Zarinara et al., 2021) in the cases of 3 or 4 treatment cycles.

Secondly, at T2, 18.5% of the sampled couples withdrew from medical treatments without achieving a biological pregnancy. Adoptive couples represent the whole of the sample withdrawing from treatments, and no childless couples were found. Therefore, in the present study, it is possible to hypothesize high levels of rejection of childless condition. The same rejection is also confirmed by the high percentage of couples pursuing treatments after 4 years (38.9%), with an average of 7 treatments (range 6–9 treatments).

These preliminary findings provided a varied portrait of the different paths infertile couples may undergo throughout after 4 years and repeated treatment cycles and highlighted one of the first key information to be carefully addressed. Indeed, multidisciplinary counseling interventions cannot bypass the ambiguity/dilemmas linked to the evidence from infertility research that, on the one side, demonstrated that the more the treatment cycles, the more the success rates increase (Malizia et al., 2009; Zarinara et al., 2021) and, on the one other side, also underlined the psychological burden of repeated treatments/treatment failures (Domar et al., 2010; Massarotti et al., 2019). In the same perspective, counseling interventions may consider that research highlighted that the choice of withdrawal and adopting or the choice of pursuing medical treatments are significantly influenced by several individual, personality and couples' relational factors (Zurlo et al., 2023) which required tailored attention.

Nonetheless, the aim of the present study was to go in-depth into the psychological health conditions of couples undergoing infertility treatments at the beginning of the path (T1) and after 4 years (T2). Therefore, responding to the Research Question of the present study (i.e., are there differences in changes over a period of 4 years in levels of psychopathological symptoms reported by male and female partners of infertile couples according to the prospective outcome groups?), data showed that the prospective outcome groups statistically differ in changes over time, underlining a significant increasing trend in psychopathological symptoms among members of couples Childless and Pursuing Treatments (CPT) and a decreasing trend among members of couples who achieved Parenthood after Successful Treatments (PST) or by Adoption (PA), mainly among men.

By examining findings from the Linear Mixed-Effect Models along with descriptive data concerning clinical levels of psychopathological symptoms a more detailed clinical insight of these findings was obtained. Indeed, despite no substantial differences emerged at T1 considering the three prospective outcome groups, findings already underlined remarkable levels of psychological suffering—mainly among women. These data, referring to the first year of treatment, confirmed that infertility diagnosis and medical treatments deeply impact the psychological health conditions of female infertile patients (El Kissi et al., 2013; Li et al., 2021; Zurlo et al., 2019).

Nonetheless, considering the changes reported from T1 to T2 by both women and men belonging to the Childless still Pursuing Treatments group (CPT), data highlighted an increase in clinical levels of Anxiety, Depression, and Paranoid Ideation. Overall, these data seem to reflect the burden of the years of repeated experiences of hope and failures related to treatments, but also the feelings of being threatened and persecuted by their condition of conceiving difficulties, on which they can perceive to have no control on (Zurlo et al., 2018, 2020b, 2023).

However, some sex specificities in clinical profiles were also found. The first data that should be highlighted refers to levels of somatization in all the sampled women, which were already alarming within T1 and even increased from T1 to T2 independently from the infertility/treatment's outcome. These data seem to suggest that the body, through somatic symptoms, represents the main channel by which infertile women may express their burden and suffering of infertility experience, even beyond the success/unsuccess of the treatments. Moreover, these findings can be also discussed considering that women go through a greater body involvement during ART treatments, which may lead to a higher risk of manifesting psychological suffering by physical symptoms. Yet, somatic symptoms reported by all the women in the present study may also be linked to the changes and challenges all the sampled women were facing at T2 (after 4 years), even in cases of achieving parenthood by ART or by adoption. Indeed, these results are in line with previous research revealing that even successful IVF mothers may experience a complex transition to parenthood with intense psychological disease (Domar, 2017; McMahon et al., 2011; Munk-Olsen and Agerbo, 2015; Sejbaek et al., 2015). In the same direction, these results may be interpreted also considering that, except for the pregnancy itself, adoptive parents undergo the same difficulties in the transition to parenthood as biological parents. They may also be subject to additional unique and potentially stressful hardships which include coping with the inability to conceive, agency evaluations of parental fitness, the uncertain wait for an eligible child, the adoption experience itself, social stigma, and potential medical, developmental, or biological problems of the adopted child (Zurlo et al., 2023).

However, still considering female partners, data also revealed an increase in scores (and in clinically relevant levels) of Obsessive-Compulsive symptoms among CPT women. This symptomatology may be also considered as related to the recourse to repeated medical treatments (Abramov et al., 2022; Tavousi et al., 2022). In this perspective, due to the significantly higher presence of Obsessive-Compulsive symptoms among CPT women, and considering the psychological risks related to long-term assisted reproductive technologies treatments, tailored counseling interventions should carefully explore individual and couples' expectations, representations and motivations potentially associated with the difficulties of considering alternative paths to achieve parenthood.

Moreover, considering male partners, only for those belonging to the Childless still Pursuing Treatments group (CPT) data revealed an increase from T1 to T2 in scores (and in clinically relevant levels) of Hostility. These findings enlightened some specificities in the expression of suffering linked to prolonged infertility experience, with men who, despite being less physically involved in treatment paths, reporting feelings of frustration, anger, and hostility (Abdullah et al., 2017).

Conversely, for men who achieved parenthood after Successful Treatments (PST) and/or by Adoption (PA), data revealed a substantial decrease in scores (and in clinically relevant levels) of Anxiety, Depression, Hostility, Interpersonal Sensitivity, and Paranoid Ideation.

These findings highlighted the positive impact of achieving parenthood by successful ART on psychological health conditions (Repokari et al., 2005), providing evidence on how the accomplishment of parenthood wishes via medical treatments may notably reduce, among men, anxious and depressive symptoms, anger, and hostility, feelings of inadequacy and interpersonal sensitivity, as well as feelings of persecution and threat (i.e., paranoid ideation). Similarly, men who achieved Parenthood by Adoption (PA) also reported a relevant decrease in clinical cases of Anxiety and Depression, with even zeroed out the clinical symptoms of Hostility, which were noteworthy alarming at the beginning of the infertility treatment (even higher than CPT couples).

Overall, findings underlined a substantial increasing trend of psychological suffering among both members of CPT couples, and a decreasing trend among PST and PA couples, even if mainly among men. Nonetheless, when exploring in detail the prevalence of clinically relevant levels of psychopathological symptoms between T1 and T2, data highlighted that the psychological health conditions of PST and PA men are significantly improved, while the psychological suffering of PST and PA women remain high and relatively stationary.

These differences in the psychological impact of both successful treatments and the alternative adoptive path deserve future research. However, these findings suggest the possibility of considering men's ability to re-adjust after infertility treatment experience as a significant resource to be enhanced within couples' counseling interventions.

Altogether, findings from the present study should be also interpreted considering the mixed evidence provided by research on the topic. Indeed, data from the present study underlined, on the one side, noteworthy high and increasing rates of somatic psychopathological symptoms among all the women belonging to the three prospective outcome groups and, on the one other side, the presence of higher and increasing psychological suffering in women and men Childless still Pursuing medical Treatments. These findings are in line with some previous comparative research (Galhardo et al., 2011; Yli-Kuha et al., 2010; Zurlo et al., 2023) and, on the opposite, are in contrast with research underlining psychopathological implications among women giving birth after ART, with even a significantly higher risk for developing psychiatric disorders (Munk-Olsen and Agerbo, 2015), mainly in terms of depression and post-partum depression (Baldur-Felskov et al., 2013; Sejbaek et al., 2015). These data may be however also due to cultural differences concerning the role of achieving parenthood in the building of individual identity (i.e., the present prospective study sample only comprised members of Italian couples) and require further investigations.

In conclusion, the present prospective study provides original evidence on psychological health conditions reported by male and female partners of infertile couples at two-time points. Findings should be used to develop tailored counseling interventions to promote psychological health and to prevent disease escalation among infertile couples after ART treatments. Furthermore, numerous aspects of the study design increase the validity of our study findings. In particular, the sample was large enough (*N* = 108 couples, i.e., 216 participants) to test our research question, the design was prospective, and the measures were psychometrically sound. Moreover, 93.9% of subjects participated in both the T1 and T2 assessments for this study, which is a high participation rate mainly given the 48-month interval between recruitment and the follow-up assessment. Finally, since the shared nature of infertility experience, to the best of our knowledge, this is the first prospective study assessing and comparing psychological health conditions in both male and female partners belonging to PST, PA, and CPT groups.

Despite these strengths, some methodological limitations should be considered. Firstly, our study population consists only of infertile couples who sought medical help, and, therefore, the findings may not generalize to other involuntary childless individuals who are not seeking ART treatments. Secondly, despite our sample being large enough to test our research question, a larger sample would allow stratifying the participants into different subgroups according to the type of infertility diagnosis or type of ART treatments and also to test other research questions exploring the potential role of individual characteristics (e.g., age, educational level, coping strategies), infertility-related characteristics (e.g., duration of infertility), situational and cultural dimensions, or relational dimensions (e.g., dyadic adjustment) influencing infertile patients' mental health. Thirdly, members of Childless Quitting Treatments (CQT) couples were not represented in our sample, and this could also limit the generalizability or comparative interpretation of the results, thus raising the need to develop further research to also address this study population. Indeed, this absence may be due to several reasons and, for example, it may be due to the self-selected nature of the sample, i.e., participants who voluntarily agreed to take part in this study could also be the one highly motivated to achieve parenthood and less likely to renounce to this desire, thus belonging to the groups who were still attempting to have a child, or who have achieved parenthood by treatment success or by adoption. Furthermore, the generalizability of research findings could also be restricted since the sample consisted of members of Italian infertile couples recruited at three Centers of Reproductive Medicine (clinic-based sample). Therefore, future studies should be designed to include couples aided at the National Healthcare Service, and—although findings could be of international interest—further research could be developed with a cross-cultural design to test the generalizability of our results beyond the Italian context.

Notwithstanding these limitations, in conclusion, this prospective study provided a greater understanding of potential changes in psychological health outcomes of both male and female infertile patients undertaking ART treatments over time. Therefore, psychological assessment and support interventions during clinical practice could address this evidence to prevent psychopathological disease escalation and promote infertile patients' wellbeing among would-be parents during and after ART treatments.

## Data Availability

The data analyzed in this study is subject to the following licenses/restrictions: The dataset will be provided upon reasonable request from the corresponding author. Requests to access these datasets should be directed to Maria Clelia Zurlo, zurlo@unina.it.
